# Normal weight obesity is associated with lower AFC and adverse IVF outcomes

**DOI:** 10.3389/fendo.2024.1332995

**Published:** 2024-02-22

**Authors:** Yangcheng Yao, Wenjuan Liu, Xiqian Zhang, Nianjun Su, Li Huang, Yingqi Nong, Xiaomin Xiao, Fenghua Liu

**Affiliations:** ^1^ Department of Obstetrics and Gynecology, The First Affiliated Hospital, Jinan University, Guangzhou, China; ^2^ Center for Reproductive Medicine, Guangdong Women and Children Hospital, Guangzhou, China

**Keywords:** body mass index, percentage of body fat, normal weight obesity, antral follicle count, reproductive outcomes

## Abstract

**Background:**

Body weight could be classified into underweight, normal weight and overweight according to percentage of body fat (%BF), and normal weight obesity (NWO) is defined as a normal BMI but a high %BF. While the impact of NWO in women fecundity remain unknow. Therefore, this study aimed to investigate the associations between %BF and reproductive outcomes among *in vitro* fertilization (IVF) women with normal BMI.

**Methods:**

A total of 469 women were included in this study and were classified into low %BF, normal %BF and high %BF according to previous study. Multivariate generalized regression models were employed to evaluate the associations of %BF with ovarian reserve parameters, IVF outcomes and early pregnancy outcomes. We further run sensitivity analyses by restricted the analysis to young women and those only with tubal factor, respectively.

**Results:**

About 32.2% of normal BMI women were misclassified according %BF, with 16.4% of them were low %BF and 15.8% were high %BF. The high %BF group had significantly lower antral follicle count (AFC) than normal %BF groups, and the AFC showed a tendency of decrease as %BF increased. In sensitivity analysis in young women, high %BF group also had significantly lower number of good-quality embryos when compared to normal %BF groups. The results expanded to all IVF outcomes when analysis restricted to tubal factor women.

**Conclusion:**

In summary, misclassifications of body weight status based on BMI are common according to %BF, and NWO is associated with adverse reproductive outcomes.

## 1 Introduction

The prevalence of overweight and obesity has globally increased during the last several decades in both men and women and both adults and children (1). Obesity is an independent risk factor for a myriad disease (e.g., type 2 diabetes, dyslipidemia, hypertension, coronary heart disease) and mortality (2). Obesity also exerts negative effects on reproduction and increases the risk of infertility (3). Epidemiological studies have demonstrated that an increased body mass index (BMI) is associated with lower ovarian responsiveness to ovulation induction, a lower number of retrieved oocytes and oocyte quality, and lower implantation, clinical pregnancy and live birth rates (4–7). The underlying mechanisms are extensive and include hormone changes, abnormal metabolism, ovulatory dysfunction, chronic inflammation and disorder, reactive oxygen species, mitochondrial dysfunction, and meiotic spindle disruption (8–10).

Obesity is defined by the WHO as abnormal or excessive fat accumulation that may impair health (11). Although BMI is a simple and commonly used index for assessing obesity, its accuracy is affected by some confounders, such as body fat-free mass, sex and age (12). Obesity misclassification is common and may lead to underestimating for health risks such as metabolic syndrome (13). Therefore, approaches for quantifying human body composition have been developed. Dual X-ray absorptiometry (DXA) is a well-accepted method for body composition assessment (e.g., bone density, fat mass, fat-free mass), which shows great consistency with MRI and CT and is regarded as a reference technique for body composition assessment in many studies (14, 15). However, DXA also has some disadvantages; for example, the machine is expensive, large, requires specialist staff and exposes the participant to radiation, which has limited its promotion.

Bioelectrical impedance analysis (BIA) devices use a harmless electrical current to assess human body composition by measuring the resistance and impedance and making predictions with its built-in algorithms (16). BIA demonstrated good agreement with DXA for fat mass and fat-free mass (17, 18), especially in the normal BMI range population (19). Studies suggest that BIA can be used as an alternative to DXA for body composition assessment due to its safety, inexpensiveness, convenience, reproducibility and efficiency (19, 20). Currently, BIA devices are widely used in clinical practice, public health, and research studies. With the application of BIA, based on the population of the National Health and Nutrition Examination Survey (NHANES) in the US, Zhu et al. developed cut-offs values for the percentage of body fat (%BF) by determining the metabolic syndrome risk equivalent to various BMI cut-off. The cut-off for %BF in women were set to 24%, 31%, 37% and 43%, which corresponded to BMI values of 18.5, 25, 30 and 35 kg/m², respectively (21).

With this %BF classification, misclassification of weight between BMI and %BF has been reported, and women are more likely to have an underestimated risk of overweight or obesity according to the BMI criteria (22). Therefore, the concept of normal weight obesity (NWO) was developed, defined as a normal BMI but high %BF (23). The associations between mismatched body weight and reproductive outcomes have rarely been researched. The present study aims to investigate the associations between %BF and reproductive outcomes among *in vitro* fertilization (IVF) women with normal BMI. We retrospectively collected and compared the clinical parameters between underweight, normal weight and overweight IVF women according to %BF.

## 2 Materials and methods

### 2.1 Study population

This retrospective study was performed at Guangdong Women and Children’s Hospital, and was approved by the institutional review board of the hospital. Women who attended the clinic of the reproductive medicine center from January 2018 to September 2020 and completed the body composition assessment were eligible for this study. The inclusion criteria were as follows: 1) women aged 20-45 years, 2) a BMI of 18.5-24.9 kg/m^2^, 3) available body composition assessment data, and 4) IVF or ICSI treatment. The exclusion criteria were as follows: 1) parental chromosomal or genetic abnormalities, 2) no eggs retrieved or oocyte cryopreservation, 3) donated oocytes, 4) attempted enrolment during a thawing cycle, and 5) a history of iatrogenic ovarian injury. The demographic information of the participants was collected from the medical system (e.g., ethnicity, smoking status, alcohol consumption and parity) and checked by clinical staff.

### 2.2 Body composition assessment

Body composition was measured by a BIA device (IOI353, HONGTAISHENG Co., Beijing, China) under the guidance of trained staff. The measurement was implemented in a standardized environment, during which the subject was instructed to remain motionless and relaxed. The analyzer measured the whole-body electrical resistance and impedance, operated through built-in algorithms, and then exported the results, including height, weight, body fat mass, %BF, degreased body weight and body water content.

### 2.3 Clinical data

The reproductive data were abstracted from the electronic medical records. Patients received the appropriate treatment at the discretion of their primary physician. The diagnosis of infertility included female factors, male factors, mix factors and unexplained reasons. Female factors included tubal factor, ovulation disorders, diminished ovarian reserve, endometriosis, and uterine factors. Male factors included semen abnormalities and coital infertility. The ovarian stimulation protocol in this study included long GnRH agonists, GnRH antagonists, luteal phase, progestin primed ovarian stimulation and mild stimulation. Reproductive data included ovarian reserve parameters (serum anti-Mullerian hormone, AMH; day 3 follicle stimulating hormone, FSH; and antral follicle count, AFC), IVF outcomes (the number of oocytes retrieved, fertilized oocytes, cleaved embryos, and good-quality embryos on day 3) and early pregnancy outcomes (implantation, biochemical pregnancy, and clinical pregnancy). Good-quality embryos were defined as grade I and II embryos according to the Cummins criteria (24).

For early pregnancy outcomes, we included only the next fresh embryo transfer cycle for the current analysis. We defined implantation as serum β-HCG ≥ 5 IU/L 12 days after embryo transfer, clinical pregnancy as the presence of at least one gestational sac on ultrasound 5 weeks after embryo transfer, and biochemical pregnancy as confirmed implantation with failure to achieve clinical pregnancy (25). Additionally, the implantation rate was defined as the number of intrauterine sacs divided by the number of embryos transferred, the clinical pregnancy rate as the number of clinical pregnancy cycles divided by the number of embryo transfer cycles, and the biochemical pregnancy rate as the number of biochemical pregnancy cycles plus the number of clinical pregnancy cycles divided by the number of embryo transfer cycles.

### 2.4 Statistical analyses

Demographic and clinical characteristics are expressed as the means ± SDs or number (%) where appropriate. Subjects were classified into low %BF (underweight), normal %BF (normal weight) and high %BF (overweight) according to cut-off values of 24% and 31% (21). The differences for baseline between three groups were tested using one-way ANOVA for continuous variable and chi-square test for categorical variable. Multivariate generalized linear regression models were used to evaluate the associations of %BF with FSH and AMH levels. Multivariate Poisson regression models were conducted to explore the associations of %BF with AFC, retrieved oocytes, fertilized oocytes, cleaved embryos, and good-quality embryos. Multivariate logistic regression models were employed to estimate the associations of %BF with implantation rate, biochemical pregnancy rate and clinical pregnancy rate. Furthermore, we included %BF as a continuous variable in the models to investigate the trends in the associations of %BF with reproductive outcomes.

Covariates were selected according to biological relevance or prior knowledge (26). For the ovarian reserve parameters, the covariates were age (continuous), BMI (continuous), ethnicity (Han, other), smoking status (ever, never), alcohol consumption (ever, never) and infertility diagnosis. For IVF outcomes, the covariates were mentioned above plus the ovarian stimulation protocol and insemination technique (IVF, ICSI). For early pregnancy outcomes, the covariates were age, BMI, ethnicity, smoking status, alcohol consumption and parity history (0, ≥ 1). Age is an important independent risk factor for adverse pregnancy outcomes (27, 28). To explore the potential effect modification of age, we ran sensitivity analyses by included women aged 20-35 years. As infertility factors may affect ovarian function, we also ran sensitivity analyses by restricting the analysis to women with tubal factor infertility. Statistical significance was indicated if the P value < 0.05. Data were analysed by Statistical Package for the Social Sciences (SPSS, version 22.0).

## 3 Results

A total of 469 subjects were included in this study, as shown in **Table 1**. The average age of the study population was 31.3 years; 77 (16.4%) of them had low %BF (< 24%), and 74 (15.8%) had high %BF (≥ 31%), according to a previous study (21). The mean AFC and number of retrieved oocytes, fertilized oocytes, cleaved embryos, and good-quality embryos on day 3 were 14.5, 12.8, 7.5, 7.4 and 5.0, respectively (**Table 2**). A total of 275 women underwent fresh embryo transfer, 142 of whom achieved clinical pregnancy.

**Table 1:** Description of the study population (N = 469).

Characteristics^a^	Total	Low %BF < 24% (N = 77, 16.4%)	Normal %BF 24-30.9% (N = 318, 67.8%)	High %BF ≥ 31% (N = 74, 15.8%)	P^b^
Age (year)	31.3 ± 4.6	29.5 ± 3.8	31.5 ± 4.7	32.0 ± 4.7	0.29
BMI (kg/m²)	22.0 ± 1.8	19.6 ± 1.0	22.1 ± 1.4	24.2 ± 0.6	< 0.001
Ethnicity					0.36
Han	452 (96.4)	75 (16.6)	307 (67.9)	70 (15.5)	
Other	17 (3.6)	2 (11.8)	11 (64.7)	4 (23.5)	
Smoking status					0.62
Ever	16 (3.4)	0 (0)	15 (93.8)	1 (6.2)	
Never	453 (96.6)	77 (17.0)	303 (66.9)	73 (16.1)	
Alcohol consumption					0.32
Ever	21 (4.5)	3 (14.3)	17 (80.9)	1 (4.8)	
Never	448 (95.5)	74 (16.5)	301 (67.2)	73 (16.3)	
Parity					0.05
0	341 (72.7)	64 (18.8)	228 (66.9)	49 (14.3)	
≥1	128 (27.3)	13 (10.2)	90 (70.3)	25 (19.5)	
Duration of infertility (years)	3.7 ± 2.7	3.4 ± 2.1	3.7 ± 2.8	3.8 ± 2.4	0.44
Infertility diagnosis					0.35
Female factors	294 (62.7)	44 (15.0)	201 (68.4)	49 (16.6)	0.82
Tubal factor	160 (54.4)	23 (14.4)	113 (70.6)	24 (15.0)	
Ovulation disorders	50 (17.0)	8 (16.0)	32 (64.0)	10 (20.0)	
Diminished ovarian reserve	49 (16.7)	10 (20.4)	29 (59.2)	10 (20.4)	
Endometriosis	12 (4.1)	1 (8.3)	8 (66.7)	3 (25.0)	
Uterine factors	23 (7.8)	2 (8.7)	16 (69.6)	5 (21.7)	
Male factors	44 (9.4)	9 (20.5)	33 (75.0)	2 (4.5)	
Mix factors	94 (20.0)	14 (14.9)	62 (66.0)	18 (19.1)	
Unexplained	37 (7.9)	10 (27.0)	22 (59.5)	5 (13.5)	

a Data are expressed as the mean ± SD or N (%).

b Data are compared by one-way ANOVA or chi-square test.

**Table 2:** Reproductive characteristics of the subjects.

Characteristics	Mean ± SD or N (%)
AFC	14.5 ± 7.8
FSH (mIU/ml)	7.6 ± 3.1
AMH (ng/ml)	4.5 ± 3.9
Total gonadotrophin (IU)	2121 ± 828
COH strategy	
Long GnRH agonist	243 (51.8)
GnRH antagonist	178 (38.0)
Luteal phase	25 (5.3)
PPOS	17 (3.6)
Mild stimulation	6 (1.3)
Insemination technique	
IVF	317 (67.6)
ICSI	152 (32.4)
Oocytes retrieved	12.8 ± 8.2
Fertilized oocytes	7.5 ± 5.3
Cleaved embryos	7.4 ± 5.2
Good-quality embryos on day 3	5.0 ± 4.3
Fresh embryo transfer	275 (58.6)
1 embryo	187 (68.0)
2 embryos	88 (32.0)
Early pregnancy outcomes	
Implantation failure	121 (44.0)
Biochemical pregnancy	12 (4.4)
Clinical pregnancy	142 (51.6)

The ovarian reserve parameters and IVF outcomes categorized by %BF are presented in **Table 3**. The mean AFCs of the low %BF, normal %BF and high %BF groups were 14.62, 14.57 and 14.11, respectively. Subjects in the high %BF group had significantly lower AFC than those in the normal %BF group. Moreover, the AFC tended to decrease as %BF increased across the three groups (P < 0.001). Regarding the remaining ovarian reserve parameters, the high %BF group showed slightly lower values than the normal and low groups, but the differences were not significant. Additionally, there were no significant differences in IVF outcome parameters among the groups.

**Table 3:** Reproductive outcomes between groups according to %BF (N = 469).

Characteristics	Low %BF (N = 77)	Normal %BF (N = 318)	High %BF (N = 74)	P1	P2	P-trend
AFC^a^	14.62 ± 7.9	14.57 ± 7.6	14.11 ± 8.2	0.10	**< 0.001**	**< 0.001**
FSH^b^	8.26 ± 3.8	7.64 ± 3.0	7.01 ± 2.4	0.80	0.92	0.82
AMH^b^	4.71 ± 3.8	4.48 ± 4.0	4.16 ± 3.6	0.66	0.33	0.70
Oocytes retrieved^c^	13.71 ± 7.9	12.44 ± 7.7	13.24 ± 10	0.40	0.59	0.70
Fertilized oocytes^c^	7.82 ± 5.2	7.45 ± 5.1	7.55 ± 6.1	0.61	0.98	0.53
Cleaved embryos^c^	7.78 ± 5.1	7.26 ± 4.9	7.39 ± 6.1	0.48	0.99	0.41
Good-quality embryos^c^	5.26 ± 4.5	4.96 ± 4.0	4.69 ± 5.0	0.92	0.94	0.98

a Compared by Multivariate Poisson regression models, adjusted for age, BMI, ethnicity, smoking status, alcohol consumption and infertility diagnosis.

b Compared by Multivariate generalized linear regression models, adjusted for age, BMI, ethnicity, smoking status, alcohol consumption and infertility diagnosis.

c Compared by Multivariate Poisson regression models, adjusted for age, BMI, ethnicity, smoking status, alcohol consumption, infertility diagnosis, ovarian stimulation protocol and insemination technique.

P1 and P2 are the P values of the low %BF group and high %BF group when compared to the normal %BF group, respectively.

P-trend means the linear trend of clinical parameters across the three groups.

Bold indicates that the comparison was significant (P < 0.05).

In the sensitivity analysis with women aged 20-35 years, the above results were also observed. Moreover, the high %BF group had a significantly lower number of good-quality embryos on day 3 than the normal %BF group (**Table 4**). In the analysis restricted to IVF women with tubal factor infertility, differences were also observed in other IVF outcomes. Women with high %BF had significantly lower numbers of retrieved oocytes, fertilized oocytes, and cleaved embryos. Furthermore, the AFC and the numbers of retrieved oocytes, fertilized oocytes, cleaved embryos, and good-quality embryos on day 3 significantly tended to decrease as %BF increased across the three groups (**Table 5**).

**Table 4:** Reproductive outcomes between groups according to %BF among women aged 20-35 years (N = 383).

Characteristics	Low %BF (N = 74)	Normal %BF (N = 251)	High %BF (N = 58)	P1	P2	P-trend
AFC^a^	14.78 ± 7.8	16.04 ± 7.6	14.58 ± 8.2	0.32	**< 0.001**	**< 0.001**
FSH^b^	8.06 ± 3.5	7.22 ± 2.2	7.19 ± 2.3	0.74	0.10	0.40
AMH^b^	4.86 ± 3.8	5.11 ± 4.2	4.49 ± 3.6	0.77	0.33	0.67
Oocytes retrieved^c^	13.89 ± 7.8	13.10 ± 7.7	12.05 ± 7.5	0.26	0.31	0.16
Fertilized oocytes^c^	7.88 ± 5.0	7.74 ± 5.1	6.95 ± 4.8	0.70	0.13	0.22
Cleaved embryos^c^	7.85 ± 4.9	7.53 ± 4.9	6.74 ± 4.8	0.49	0.12	0.15
Good-quality embryos^c^	5.27 ± 4.4	5.29 ± 4.2	3.90 ± 3.5	0.53	**0.017**	0.31

a Compared by Multivariate Poisson regression models, adjusted for age, BMI, ethnicity, smoking status, alcohol consumption and infertility diagnosis.

b Compared by Multivariate generalized linear regression models, adjusted for age, BMI, ethnicity, smoking status, alcohol consumption and infertility diagnosis.

c Compared by Multivariate Poisson regression models, adjusted for age, BMI, ethnicity, smoking status, alcohol consumption, infertility diagnosis, ovarian stimulation protocol and insemination technique.

P1 and P2 are the P values of the low %BF group and high %BF group when compared to the normal %BF group, respectively.

P-trend means the linear trend of clinical parameters across the three groups.

Bold indicates that the comparison was significant (P < 0.05).

**Table 5:** Reproductive outcomes between groups according to %BF among women with tubal factor infertility (N = 160).

Characteristics	Low %BF (N = 23)	Normal %BF (N = 113)	High %BF (N = 24)	P1	P2	P-trend
AFC^a^	13.95 ± 4.6	14.43 ± 5.6	13.17 ± 5.5	0.55	**0.009**	**0.028**
FSH^b^	7.96 ± 2.0	7.33 ± 1.8	6.96 ± 2.0	0.17	0.52	0.19
AMH^b^	3.49 ± 1.8	4.12 ± 3.0	4.33 ± 2.6	0.25	0.38	0.19
Oocytes retrieved^c^	17.78 ± 7.9	13.81 ± 7.0	11.04 ± 7.1	**< 0.001**	**< 0.001**	**< 0.001**
Fertilized oocytes^c^	9.96 ± 4.9	8.58 ± 4.5	6.83 ± 5.2	**0.014**	**< 0.001**	**< 0.001**
Cleaved embryos^c^	9.83 ± 4.8	8.32 ± 4.3	6.54 ± 5.2	**0.008**	**< 0.001**	**< 0.001**
Good-quality embryos^c^	6.22 ± 4.5	5.68 ± 3.8	4.13 ± 4.4	0.11	**0.003**	**0.003**

a Compared by Multivariate Poisson regression models, adjusted for age, BMI, ethnicity, smoking status, alcohol consumption and infertility diagnosis.

b Compared by Multivariate generalized linear regression models, adjusted for age, BMI, ethnicity, smoking status, alcohol consumption and infertility diagnosis.

c Compared by Multivariate Poisson regression models, adjusted for age, BMI, ethnicity, smoking status, alcohol consumption, infertility diagnosis, ovarian stimulation protocol and insemination technique.

P1 and P2 are the P values of the low %BF group and high %BF group when compared to the normal %BF group, respectively.

P-trend means the linear trend of clinical parameters across the three groups.

Bold indicates that the comparison was significant (P < 0.05).

For the early pregnancy outcomes, compared with the low %BF and normal %BF groups, the high %BF group had a lower implantation rate, biochemical pregnancy rate and clinical pregnancy rate, but the differences were not significant (**Table S1**). There were also no significant differences in implantation rate, biochemical pregnancy rate or clinical pregnancy rate among the three groups in the sensitivity analyses (**Tables S2, S3**).

## 4 Discussion

In this study of 469 women with normal BMI (18.5-24.9 kg/m^2^), a total of 151 subjects (32.2%) were misclassified according to %BF, with 16.4% of them being underweight (%BF < 24%) and 15.8% being overweight (%BF ≥ 31%). Consistent with our results, Kim et al. found that 28.6% (283 of 989) of normal BMI women (18.5-24.9 kg/m^2^) were overweight according to %BF (%BF ≥ 31%), but the proportion considered underweight was not estimated (22). Peterson et al. reported that nearly half of women who were misclassified as normal weight (BMI < 25 kg/m^2^) were actually obese according to %BF (≥ 35%) (13). These results demonstrate that misclassification based on BMI criteria is common. The accuracy is affected by the BMI and age; as the BMI increases, the sensitivity decreases and the specificity increases, and accuracy also decreases with increasing age (29). The mismatch rate in the previous studies was much higher than that in the present study, which may be due to the relatively lower mean BMI of the study population in this research.

We found that the AFC tended to decrease across the three groups as %BF increased, and women with high %BF had a significantly lower AFC than normal %BF women; this result persisted in the sensitivity analysis. Moreover, the decreasing tendency expanded to the IVF outcomes in the sensitivity analysis of women with tubal factor infertility (the numbers of retrieved oocytes, fertilized oocytes, cleaved embryos, and good-quality embryos on day 3). In Kim’s research, no difference was found between %BF normal weight (BMI < 25 kg/m^2^, %BF < 31%) and %BF overweight (BMI < 25 kg/m^2^, %BF ≥ 31%) women for AFC or the number of oocytes retrieved (22). This difference may be attributed to the differences in the study population, e.g., race, region, age, and study design. For the early pregnancy outcomes (i.e., implantation rate, biochemical pregnancy rate and clinical pregnancy rate), although decreasing trends were observed as %BF increase, no significance were reached. In line with our results, a previous study also reported no differences in pregnancy outcomes among IVF women stratified by %BF (30).

Obesity has been reported to impair ovarian responsiveness and is associated with lower ovarian reserve, smaller oocyte size, a lower oocyte yield, impaired oocyte quality, and suboptimal pregnancy outcomes (31–34). Obesity induces metabolic disorders, chronic low-grade inflammatory status and hormone alterations (e.g., higher levels of leptin, insulin, androgen, estrogen), which could further impair ovarian folliculogenesis (35–38). Obesity has also been associated with the altered follicular fluid, the critical environment for oocyte development and granulosa cell steroidogenesis, with elevated concentrations of leptin, insulin, triglycerides, inflammation markers (e.g., lactate and C-reactive protein), and oxidative stress (37, 39–41). Elevated levels of free fatty acids in follicular fluid are correlated with low-grade cumulus-oocyte complexes and low-quality oocytes (42). Excess free fatty acids can also induce mitochondrial and endoplasmic reticulum stress by increasing reactive oxygen species (43). In addition, obesity also appears to alter the meiotic spindles, mitochondrial distribution and function in the oocyte (10, 44). In contrast, a lower %BF generally indicates more physical activity, which may have beneficial effects for reproductive health in women (45).

The results of sensitivity analysis suggest that the associations of %BF with reproductive outcomes may modified by age and infertility diagnosis. High %BF group had less good-quality embryos on day 3 than the normal %BF group in younger women. Age is a critical independent risk factor for ovarian function, oocyte developmental potential drops in advanced age women (28), which may cover the associations of %BF with reproductive outcomes. This was also observed in sensitivity analysis of women with tubal factor infertility. Tubal factor includes hydrosalpinx, fallopian tube obstruction, and so on. Women with hydrosalpinx were generally undergoing surgical treatment before IVF. Therefore, the tubal factor was mainly included fallopian tube obstruction in the present study. And they were chosen for sensitivity analyses because tubal factor is the most common reason of women infertile, with largest number of subjects among these subgroups. The number of infertile women diagnosed with ovulation disorders, diminished ovarian reserve, endometriosis, mix factors and unexplained factors were lower, and was associated with lower oocyte quality and embryo developmental potential (46–48).

Body fat is a storage site for excessive energy, and obesity is associated with increased serum free fatty acid levels (49). Adipose tissue is also an endocrine organ and involved in coordinating a variety of biological processes including energy metabolism, neuroendocrine function, and immune function (50). Adipose tissue dysfunction is associated with insulin resistance, hyperglycemia, dyslipidemia, and hypertension (51). The cut-offs values for %BF were determined based on metabolic syndrome risk (21). For NWO individuals, although their BMI were in normal range, the body fat was exceeded and may have induce health risks. It has been reported that NWO increased risk for abnormal blood glucose, cardiometabolic morbidity and mortality (52, 53). Our research further suggests that the excessive body fat may threat to reproductive health. Metabolic alterations in serum were reflected in the follicular fluid, which is critical for oocyte development (54). Elevated levels of free fatty acids in follicular fluid could alter the granulosa cells functions by affecting steroidogenesis, proliferation, and apoptotic processes (55), affect oocytes maturation and developmental competence via induce mitochondrial dysfunction and endoplasmic reticulum stress (56). Infertility has become an ongoing reproductive health problem around the world (57), the potential adverse effect of obesity has rising concern and more and more reproductive centers are setting weight management clinics (32). However, currently it mainly serves the obese population or women with PCOS. Our research suggests that NWO infertility women may also benefit from weight management. Therefore, we recommend a body composition assessment for all new infertility patients if permit.

This study has several limitations. The first was its retrospective design, and the stringent inclusion criteria and the exclusion of candidates may have introduced bias in the study population. Second, the sample size was relatively small, especially in the sensitivity analysis for early pregnancy outcomes. Third, we included only women who underwent fresh embryo transfer for early pregnancy outcome analysis due to data limitations, and since %BF has no associations with early pregnancy outcomes, body composition assessment may have limited benefits. Despite these limitations, by measuring body composition, we found that NWO was common in women, and a higher %BF was negatively associated with AFC and IVF outcomes. To our knowledge, this is the first study to reseal these findings.

## 5 Conclusions

Our data suggest that the classification of normal weight according to BMI may be inaccurate according to %BF, and NWO is associated with adverse reproductive outcomes. Reducing body fat may benefit to the reproduction among normal BMI women. More research is required to further validate the findings of this study.

## Data availability statement

The data analyzed in this study is subject to the following licenses/restrictions: the dataset analysed during the current study are not publicly available because they contain multiple sensitive information. Data are however available only upon reasonable and necessary request. Requests to access these datasets should be directed to Fenghua Liu, liushine2006@163.com.

## Ethics statement

The studies involving humans were approved by the Ethics Committee of Guangdong Women and Children Hospital. The studies were conducted in accordance with the local legislation and institutional requirements. Written informed consent for participation was not required from the participants or the participants’ legal guardians/next of kin because this research is a retrospective study.

## Author contributions

YY: Conceptualization, Formal analysis, Writing – original draft. WL: Investigation, Resources, Writing – original draft. XZ: Data curation, Writing – review & editing. NS: Writing – review & editing. LH: Resources, Writing – original draft. YN: Investigation, Writing – review & editing. XX: Supervision, Writing – review & editing. FL: Funding acquisition, Resources, Writing – review & editing.
