# Supplementary material for: Normal weight obesity is associated with lower AFC and adverse IVF outcomes
Source: Front Endocrinol (Lausanne). 2024 Feb 22;15:1332995. doi: 10.3389/fendo.2024.1332995 (PMC10917978; doi:10.3389/fendo.2024.1332995)
Supplement: Supplementary file 1 [file DataSheet_1.docx]

**Supplementary Material**

**Normal weight obesity is associated with lower AFC and adverse IVF outcomes**

Yangcheng Yao, Wenjuan Liu, Xiqian Zhang, Nianjun Su, Li Huang, Yingqi Nong, Xiaomin Xiao, Fenghua Liu.

**Table of Content**

**Table S1**. Pregnancy outcomes between groups according to %BF.

**Table S2**. Pregnancy outcomes between groups according to %BF among women aged 20-35 years.

**Table S3**. Pregnancy outcomes between groups according to %BF among women with tubal factor infertility.

| **Table S1**. Pregnancy outcomes between groups according to %BF (N = 275). | | | | | | |
| --- | --- | --- | --- | --- | --- | --- |
| Characteristics^a^ | Low %BF  (N = 45,  16.4%) | Normal %BF  (N = 193, 70.2%) | High %BF  (N = 37, 13.4%) | P1 | P2 | P-trend |
| Implantation rate | 56.9% | 47.7% | 40.4% | 0.91 | 0.46 | 0.65 |
| Biochemical pregnancy rate | 60.0% | 56.5% | 48.6% | 0.67 | 0.30 | 0.70 |
| Clinical pregnancy rate | 55.6% | 51.8% | 45.9% | 0.96 | 0.32 | 0.54 |
| ^a^ Compared by Multivariate logistic regression models, adjusted for age, BMI, ethnicity, smoking status, alcohol consumption and parity history. | | | | | | |
| P1 and P2 are the P values of the low %BF group and high %BF group when compared to the normal %BF group, respectively. | | | | | | |
| P-trend means the linear trend of clinical parameters across the three groups. | | | | | | |

| **Table S2**. Pregnancy outcomes between groups according to %BF among women aged 20-35 years (N = 232). | | | | | | |
| --- | --- | --- | --- | --- | --- | --- |
| Characteristics^a^ | Low %BF  (N = 45, 19.4%) | Normal %BF  (N = 157, 67.7%) | High %BF  (N = 30, 12.9%) | P1 | P2 | P-trend |
| Implantation rate | 56.9% | 53.9% | 48.8% | 0.68 | 0.78 | 0.94 |
| Biochemical pregnancy rate | 60% | 61.8% | 56.7% | 0.50 | 0.52 | 0.92 |
| Clinical pregnancy rate | 55.6% | 56.1% | 56.7% | 0.84 | 0.81 | 0.99 |
| ^a^ Compared by Multivariate logistic regression models, adjusted for age, BMI, ethnicity, smoking status, alcohol consumption and parity history. | | | | | | |
| P1 and P2 are the P values of the low %BF group and high %BF group when compared to the normal %BF group, respectively. | | | | | | |
| P-trend means the linear trend of clinical parameters across the three groups. | | | | | | |

| **Table S3**. Pregnancy outcomes between groups according to %BF among women with tubal factor infertility (N = 101). | | | | | | |
| --- | --- | --- | --- | --- | --- | --- |
| Characteristics^a^ | Low %BF  (N = 13, 12.9%) | Normal %BF  (N = 73, 72.3%) | High %BF  (N = 15, 14.8%) | P1 | P2 | P-trend |
| Implantation rate | 42.9% | 42.9% | 52.4% | 0.97 | 0.93 | 0.93 |
| Biochemical pregnancy rate | 46.2% | 50.7% | 66.7% | 0.63 | 0.51 | 0.43 |
| Clinical pregnancy rate | 38.5% | 43.8% | 66.7% | 0.97 | 0.40 | 0.54 |
| ^a^ Compared by Multivariate logistic regression models, adjusted for age, BMI, ethnicity, smoking status, alcohol consumption and parity history. | | | | | | |
| P1 and P2 are the P values of the low %BF group and high %BF group when compared to the normal %BF group, respectively. | | | | | | |
| P-trend means the linear trend of clinical parameters across the three groups. | | | | | | |
